# Regulation of miR-30b in cancer development, apoptosis, and drug resistance

**DOI:** 10.1515/biol-2022-0017

**Published:** 2022-02-28

**Authors:** Zhen-Jie Fu, Yan Chen, Yu-Qin Xu, Mei-Ai Lin, Hang Wen, Yi-Tao Chen, Pei-Lei Pan

**Affiliations:** School of Life Sciences, Zhejiang Chinese Medical University, No. 548 Binwen Road, Binjiang District, Hangzhou 310053, China

**Keywords:** molecular mechanism, miR-30b, signaling pathways, cancer development, drug resistance

## Abstract

miR-30b, which is encoded by the gene located on chromosome 8q24.22, plays an important role in a variety of diseases. In most types of tumors, miR-30b significantly inhibits the proliferation, migration, and invasion of cancer cells through the regulation of target genes. Moreover, miR-30b can inhibit the PI3K/AKT signaling pathway through the regulation of EGFR, AKT, Derlin-1, GNA13, SIX1, and other target genes, thus inhibiting the EMT process of tumor cells and promoting apoptosis. In addition, miR-30 plays a significant role in alleviating drug resistance in tumor cells. Although the use of miR-30b as a clinical diagnostic indicator or anticancer drug is still facing great difficulties in the short term, with the deepening of research, the potential application of miR-30b is emerging.

## 1 Introduction

miRNA is a group of noncoding short sequence single-stranded RNAs (ssRNAs) that attenuate or eliminate the function of downstream genes by targeting the 3′-untranslated region (3′-UTR) structure of mRNA and indirectly regulate physiological and pathological states [1]. Since the first miRNA was discovered in *Caenorhabditis elegans* in 1993, hundreds of different miRNAs have been identified in humans in the past 30 years [2]. In mammals, the synthesis of miRNA is mainly divided into two steps. The first step occurs in the nucleus. The miRNA genes are first transcribed by RNA polymerase II [3] to produce primary microRNAs (pri-miRNAs), and then pri-miRNAs are processed by the Drosha-DGCR8 complex into precursor miRNAs (pre-miRNAs) [4]. Finally, the pre-miRNAs are exported to the cytoplasm via the Exportin-5 (Exp5), an RNA-dependent transport receptor transfected with importin [5,6]. The second step of processing occurs in the cytoplasm, where RNA enzymes III endonuclease and Dicer microRNAs before cutting into about 22nt long mature microRNA [7,8]. Although miRNAs are mainly distributed in the cytoplasm, several miRNAs can be repositioned to the nucleus by the miRISC shuttle [9].

A large number of studies have shown that miRNAs play a key role in many regulatory pathways such as body development, cell differentiation, cell apoptosis, metabolism, and signal transduction [10,11]. As a wide range of gene expression regulators, miRNAs, and their targets constitute a complex regulatory network [12]. On the one hand, a single miRNA can bind to one or more mRNA targets; on the other hand, multiple miRNAs can synergistically control a single mRNA target [13]. Therefore, even a single miRNA is also involved in a wide range of functional and metabolic pathways, which can directly or indirectly affect the expression of multiple downstream target genes and their corresponding biological functions. To date, miRNAs are involved in regulating the expression of more than one-third of the human protein-coding genes [14,15]. Previous studies have found that more than half of nematode miRNAs have sequence homology with human-expressed miRNAs [16]. These studies indicate that miRNAs are an important group of post-transcriptional regulators.

miR-30b, which is encoded by the gene located on chromosome 8q24.22, is a member of the miR-30 family consisting of five members (miR-30a, miR-30b, miR-30c, miR-30d, and miR-30e) and plays an important role in a variety of diseases [17]. The miR-30b sequence is highly conserved in the evolution of different species. Over the years, numerous literature reports have emphasized that miR-30b plays a crucial role in regulating key signaling pathways. The abnormal expression of miR-30b under various pathological conditions has attracted increasing attention. This article collects, collates, and summarizes the literature related to miR-30b in the past 15 years and discusses the relationship between miR-30b and tumor, apoptosis, and drug resistance.

## 2 Role of miR-30b in cancer development

Proliferation, migration, and invasion are the most basic features of the cancer development process. Since the targets of miR-30b contain numerous proteins related to proliferation, migration, and invasion, the dysregulation of miR-30b expression leads to the uncontrolled development of tumor cells (Table 1).

**Table 1:** The regulation of miR-30b on cancer development

Cell type and physiology	Target	The functions involved
Non-small-cell lung cancer [18]	Cthrc1	Invasion and migration
Osteosarcoma cells [19]	ATG5	Proliferation, invasion, and autophagy
Esophageal cancer [20]	HOXA1	Proliferation, migration, and invasion
Esophageal squamous cell carcinoma [21]	HOXA13	Proliferation
Gallbladder carcinoma [22]	NT5E	Proliferation, migration, and invasion
Glioblastoma [23]	PRRT2	Proliferation, migration, and invasion
Colorectal cancer [24]	KITENIN	Migration and invasion
Colorectal cancer [25]	SIX1	Migration and invasion
Hepatocyte [27]	Snail	EMT
Gastric cancer [28]	EIF5A2	EMT
Breast cancer, CC [29]	Drelin-1	PI3K/AKT-EMT
Renal cell carcinoma [30]	GNA13	PI3K/AKT-EMT

In non-small cell lung cancer, it was shown that miR-30b directly binds to the 3′-UTR of collagen triple helix repeat containing 1 (Cthrc1), which is closely related to lymph node metastasis [18]. In osteosarcoma cells, autophagy protein 5 (ATG5) not only participates in the formation of autophagosomes but also promotes cancer cell proliferation and invasion. Therefore, miR-30b targeting ATG5 not only participates in the regulation of autophagy but also inhibits the proliferation and invasion of cancer cells [19]. In the research related to esophageal squamous carcinoma, homeobox protein Hox-A1 (HOXA1) and homeobox protein Hox-A13 (HOXA13) were confirmed as the target genes of miR-30b. The sponge effect of reintroduced and long-chain non-coding RNA (lncRNA) HOTTIP was used to weaken the targeted inhibition of miR-30b on HOXA1 and HOXA13 and to improve the protein expression level. As a result, the inhibition effect of miR-30b on the proliferation, metastasis, and invasion of cancer cells was reversed [20,21]. In gallbladder carcinoma, miR-30b inhibits the proliferation, metastasis, and invasion of cancer cells by targeting ecto-5′-nucleotidase (NT5E) [22] and proline-rich transmembrane protein 2 (PRRT2) [23]. In several colorectal cancer cell lines, miR-30b suppressed the migration and invasion via modulation of KAI1 C-terminal interacting tetraspanin (KITENIN) expression [24].

**Figure 1:**
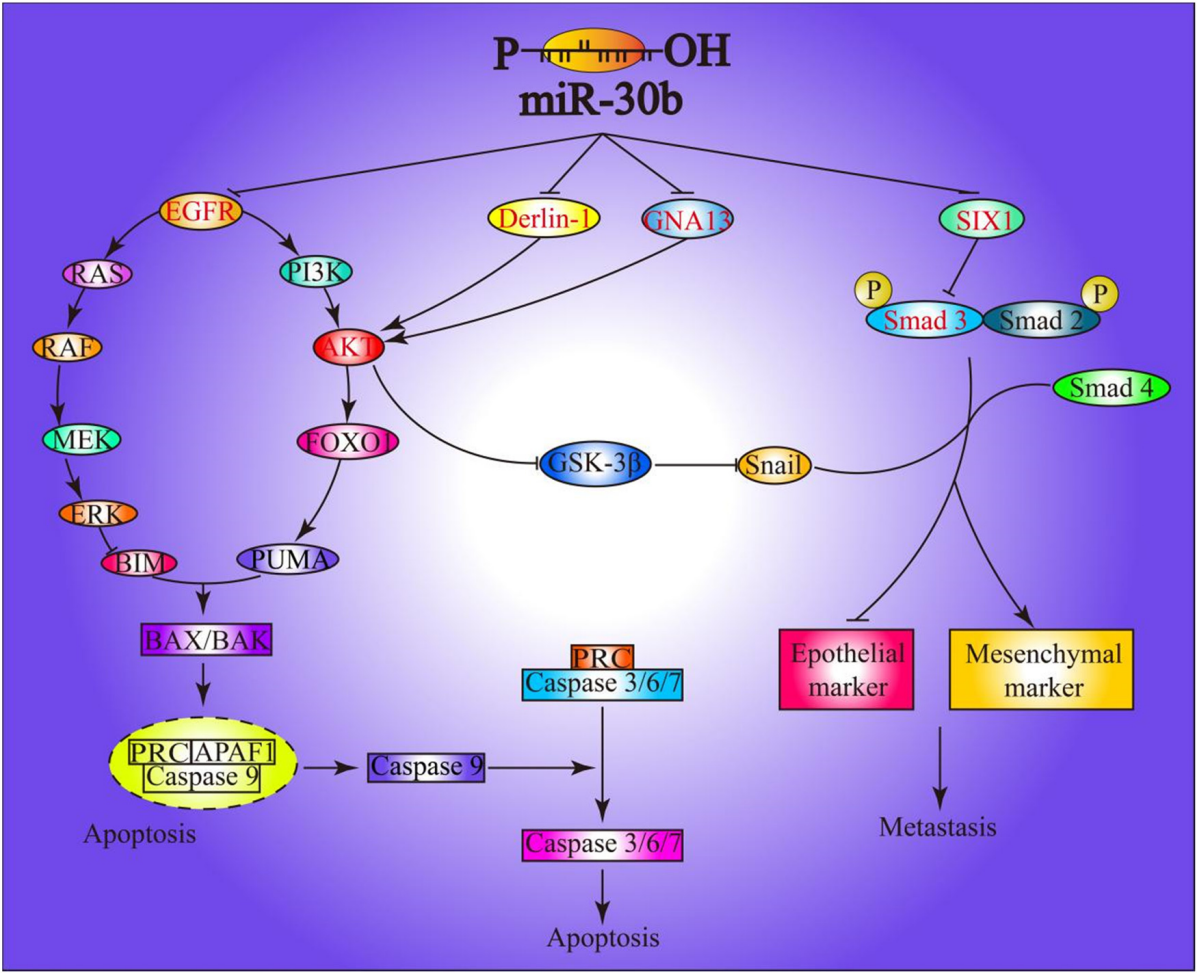
The regulatory network of miR-30b on EMT and apoptosis.

In addition, in colorectal cancer and pancreatic cancer, other studies have found that miR-30b targeted SIX homeobox 1 (SIX1) and snail, which were EMT (epithelial–mesenchymal transition)-promoting genes [25]. EMT is currently regarded as a pathological process that leads to tumor progression. In the process of malignant tumor evolution, EMT enables tumor cells to metastasize and invade and may also enable tumor cells to escape from certain factors-induced apoptosis. Therefore, in the study of tumors, the occurrence of EMT predicts the malignant progression of tumors. Studies have shown that miR-30b can form significant E-cadherin depletion and vimentin increase by targeting EIF5A2 in gastric cancer, which are signature molecules of EMT [26]. Moreover, snail, a key transcriptional activator of EMT, is also a direct target of miR-30b [27]. The PI3K-AKT-MTOR signaling pathway is an EMT activation pathway and functions by inhibiting the EMT suppressor GSK-3β (Figure 1).

Derlin-1 is a transmembrane protein located in the internal parasitic network, which is highly expressed in various tumors and plays an important role in tumorigenesis. In breast cancer, miR-30b can directly target Derlin-1 for negative regulation, and the loss of Derlin-1 inhibits the phosphorylation of AKT. Therefore, a low miR-30b expression level leads to abnormal PI3K/AKT signaling pathway activation, resulting in uncontrolled cell proliferation and invasion [28]. In addition, in cervical cancer (CC) studies, the downregulation of Derlin1 affects the activation of the AKT/mTOR signaling pathway, and the absence of Derlin1 can inhibit the expression of p-AKT and p-mTOR [29]. Additionally, miR-30b inhibits EMT in renal cell carcinoma by negatively regulating the guanine nucleotide-binding protein subunit alpha-13 (GNA13) protein expression level [30].

## 3 miR-30b regulates apoptosis

Apoptosis is an ordered cell death process that occurs in physiological and pathological conditions. Tumor necrosis factor-related apoptosis-inducing ligand (TRAIL) is a promising pro-apoptotic protein that can rapidly induce exogenous apoptosis of tumor cells without affecting normal cells. As a homologous trimer, TRAIL binds to the homologous death receptor 4 (DR4) or DR5 on the surface of target cells, resulting in the recruitment of related proteins of adaptor protein FAS with death domain (FADD), which assemble and activate caspase-8. Activation of caspase-8 leads to activation of caspase-3, which initiates apoptosis and causes cell death. In glioblastoma, caspase-3 was identified as one of the target genes of miR-30b, so overexpression of miR-30b causes apoptotic resistance [31]. In addition, miR-30b was found to inhibit the EGFR pathway by targeting EGFR in non-small-cell lung cancer, thus causing inhibition of Bax and caspase-3 [32].

PAI-1 is another target gene of miR-30b found in gastric cancer by bioinformatics analysis and western blot. It was shown that PAI-1 overexpression could counteract the effect of promoting apoptosis by miR-30, and ectopic expression of miR-30b had a similar promoting-apoptosis effect compared with silencing PAI-1 expression. PAI-1, as an inhibitor of AKT, is significantly overexpressed in gastric cancer tumors, resulting in inhibition of Bax, caspase-3, and caspase-9 expressions, thus forming apoptotic resistance [33].

## 4 Regulatory role of miR-30b in cellular drug resistance

The study on the drug resistance mechanism is the basic research on the clinical treatment of tumor drug resistance, and the function of miR-30b to regulate the expression of drug-sensitive proteins in cells through target genes has been confirmed. For example, in the study on the mechanism of trastuzumab resistance in the treatment of breast cancer, it was found that the expression of miR-30b was significantly upregulated in the sensitive strain BT474wt and the survival rate was decreased by 60%. CCNE2 is a cell cycle regulation gene, which is highly expressed in the sensitive strain BT474r and low in acquired drug-resistant cell strain BT474wt. miR-30b can precisely target CCNE2. By upregulating miR-30b and negatively regulating CCNE2 expression, HER2 + breast cancer has reduced resistance to Trastuzumab [34].

In non-small-cell lung cancer (NSCLC) studies, it has been found that miR-30b combined with a low dose rate (LDR) can inhibit the overexpression of PAI-1, effectively controlling the negative control effect of PAI-1 on the efficacy of LDR in the treatment of tumors. AKT and ERK are downstream survival signals of PAI-1, and inhibiting PAI-1 can reduce the phosphorylation of AKT and ERK. Inhibition of PAI-1 decreased the survival rate and EMT progression of NSCLC cells. In a xenograft mouse model with miR-30b as the carrier, low-dose radiation therapy and radiotherapy were shown to effectively reduce tumor growth and invasivity.

In addition to the aforementioned studies on the mechanism of action of miR-30b in the treatment of breast cancer and NSCLC with Trastuzumab, miR-30b has also been found to have a regulatory effect on drug resistance in some diseases but the specific mechanism has not been clearly explained. For example, in studies on the mechanism of drug resistance in gefitinib treatment for NSCLC, it was found that the expression of miR-30b was downregulated after treatment with MET inhibitors, and the sensitivity of NSCLC to gefitinib was increased [35,36].

## 5 Discussion

In recent years, the regulatory role of miRNAs in many diseases has attracted wide attention. The imbalance of miR-30b especially plays an important role in the occurrence and development of tumors and might be a potential target for tumor diagnosis and treatment, among which it has been reported as a biomarker for gastric cancer, breast cancer, and liver cancer. With the continuous advancement of sequencing technology, the understanding of miR-30b is increasingly comprehensive and in-depth, and the feasibility and trend of using miR-30b as a diagnostic and prognostic marker for a variety of diseases as well as the application of miR-30b in the field of disease treatment through knockdown and overexpression medical technologies are more and more prominent. However, there are still great difficulties and challenges from basic research to clinical application. The overall background of microRNA-based therapeutics is still in its infancy, and the distribution of miR-30b target genes are involved in a wide range of functions. Therefore, in the future, on the one hand, the mechanism of action between miR-30b and target genes needs to be further elaborated. On the other hand, how to achieve accurate regulation of miR-30b may be the key to its clinical application. Although the use of miR-30b as a clinical diagnostic indicator or anticancer drug is still facing great difficulties in the short term, with the deepening of research, the potential application of miR-30b will emerge.
